# Second cancer risk after radiation therapy of ependymoma using the flattening filter free irradiation mode of a linear accelerator

**DOI:** 10.1002/acm2.12438

**Published:** 2018-08-19

**Authors:** Judit Alvarez Moret, Tina Obermeier, Fabian Pohl, Rainer Loeschel, Oliver Koelbl, Barbara Dobler

**Affiliations:** ^1^ Department of Radiotherapy Regensburg University Medical Center Regensburg Germany; ^2^ Department of Computer Science and Mathematics University of Applied Sciences, OTH Regensburg Regensburg Germany

**Keywords:** children, EAR, ependymoma, flattening filter free, radiation therapy, second cancer risk

## Abstract

Pediatric patients suffering from ependymoma are usually treated with cranial or craniospinal three‐dimensional (3D) conformal radiotherapy (3DCRT). Intensity‐modulated techniques spare dose to the surrounding tissue, but the risk for second malignancies may be increased due to the increase in low‐dose volume. The aim of this study is to investigate if the flattening filter free (FFF) mode allows reducing the risk for second malignancies compared to the mode with flattening filter (FF) for intensity‐modulated techniques and to 3DCRT. A reduction of the risk would be advantageous for treating pediatric ependymoma. 3DCRT was compared to intensity‐modulated radiation therapy (IMRT) and volumetric‐modulated arc therapy (VMAT) with and without flattening filter. Dose–volume histograms (DVHs) were compared to evaluate the plan quality and used to calculate the excess absolute risk (EAR) to develop second cancer in the brain. Dose verification was performed with a two‐dimensional (2D) ionization chamber array and the out‐of‐field dose was measured with an ionization chamber to determine the EAR in peripheral organs. Delivery times were measured. Both VMAT and IMRT achieved similar plan quality in terms of dose sparing in the OAR and higher PTV coverage as compared to 3DCRT. Peripheral dose in low‐dose region, which is proportional to the EAR in organs located in this region, for example, gonads, bladder, or bowel, could be significantly reduced using FFF. The lowest peripheral EAR and lowest delivery times were hereby achieved with VMAT_FFF_. The EAR calculated based on DVH in the brain could not be reduced using FFF mode. VMAT_FFF_ improved the target coverage and homogeneity and kept the dose in the OAR similar compared to 3DCRT. In addition, delivery times were significantly reduced using VMAT_FFF_. Therefore, for radiotherapy of ependymoma patients, VMAT_FFF_ may be considered advantageous for the combination of Elekta Synergy linac and Oncentra External Beam planning system used in this study.

## 1 INTRODUCTION

The standard therapy for childhood ependymoma is surgery followed by adjuvant radiation.^1^, ^2^ Historically, cranial or craniospinal irradiation was the standard treatment after surgery^3^ in order to assure that the volume adjacent to the target receives adequate dose and consequently assure local control. However, recent publications have shown that conformal, intensity‐modulated (IMRT) and proton‐beam radiotherapy can achieve similar local control rates to those published in historical publications without an increased risk of marginal failure.^4^, ^5^, ^6^, ^7^

An advantage of IMRT over three‐dimensional (3D) conformal standard technique is to spare dose in surrounding normal tissues. However, a concern about the IMRT treatment is that the volume that receives a low dose can be significantly higher compared to those volumes for conventional technique. This might increase the risk for second malignancies, which is a very important issue with regard to the life expectancy of these pediatric patients.^8^

A recent development in the linear accelerator technology is the irradiation without a flattening filter in the beam path to increase dose rate and reduce beam‐on time.^9^ A reduction of the treatment time would represent an additional advantage, especially in the treatment of pediatric patients. The flattening filter produces scatter radiation; therefore, its removal has the positive effect of reducing the out‐of‐field dose,^10^ which may lead to reduced second cancer risk as it has been shown for the treatment of breast cancer in a previous study.^11^ To our knowledge, no reports about second cancer risk have been published for ependymoma. The excess absolute risk (EAR) of developing a second cancer after exposure to radiation (EAR) can be estimated from dose–volume histograms (DVHs) based on biological models which are fitted to data of atomic bomb survivors and Hodgkin patients treated with radiation therapy.^12^, ^13^

The purpose of our study was therefore to investigate whether the flattening filter free mode (FFF) is advantageous with respect to the second cancer risk, plan quality, and delivery time after radiation therapy of ependymoma as compared with the flat beam mode (FF).

## 2 MATERIALS AND METHODS

### 2.A Patients and treatment planning

Computed tomography (CT) data of 11 pediatric patients with ependymoma were selected from our treatment database for a retrospective planning study. The age of the patients ranged from 22 months to 15 yr with a mean of 6.5 yr and a median of 4.6 yr. All patients were completely resected and without metastases. The irradiation approach was used in this planning study in the same way as in the HIT Interim Register for children under 4 yrs^14^ (dose per fraction 1.8 Gy, total dose 50.4 Gy). HIT stands for brain tumors in children. The precursor was the HIT 2000 study, a multicenter therapy optimization study, which provided optimal and risk‐adapted therapy nationwide in Germany and Austria for patients up to the age of 21 with an intracranial localized medulloblastoma, PNET, or ependymoma. Following the completion of the HIT 2000 study, the HIT Interims Register study was launched to bridge the period until the relaunch of the study with the same standard of quality.

The patients were in supine position and an individual thermoplastic facial mask was created. The planning CT was then performed in 4 mm layer thickness from occiput to the third cervical vertebral body in a multislice CT Siemens^®^ Somatom Sensation Open. The planning CT was imported into the planning system and it was fused with the magnetic resonance tomography (T1‐weighted, contrast‐enhanced MRI) before and after surgery (MRI equipment: 1.5T Siemens^®^ Magnetom Avanto and Siemens^®^ Magnetom Symphony). The planning target volume (PTV) was the expanded tumor region, which means the former tumor region (GTV = gross tumor region), including two centimeter of automatically generated safety margin taking into account anatomical limits or rearrangement of brain tissue into the resection area after surgery. Dose threshold was based on the tolerance doses for organs at risk (OAR) of the protocol of Radiation Therapy Oncology Group Trial 0225.^15^ Dose to the normal tissue (patient outline excluding the PTV) should be kept as low as possible to minimize the risk of second cancer induction.^8^

Treatment planning was performed with Oncentra External Beam^®^4.5.2 with collapsed cone dose calculation algorithm for a Synergy linear accelerator with Agility™ head (Elekta AB, Stockholm, Sweden). Five treatment plans with 6 MV photons were created for each patient: step and shoot IMRT and VMAT plans both with and without flattening filter (IMRT_FF_, IMRT_FFF_, VMAT_FF_, and VMAT_FFF_) and a conventional five‐field 3D‐plan (3DCRT). IMRT plans consisted of nine coplanar beams (20°, 60°, 95°, 140°, 180°, 220°, 265°, 300°, and 340°). The VMAT plans were performed with a single full rotation with gantry spacing between two control points of 4°. Dose–volume objectives (DVO) used for VMAT and IMRT optimization are listed in Table 1. DVOs to the OAR were set to values which could be achieved in 3DCRT. Identical DVO were used for optimization of all plans. The dose calculation was performed with a grid size of 3 mm and the dose was normalized to the average dose in the PTV. All plans were accepted for treatment by a radiation oncologist.

**Table 1:** DVO used for optimization and tolerance dose for each structure

Structure	Type	Objective	Dose (Gy)	Weight	Distance (cm)	Tolerance dose^15^ (Gy)
PTV	Target	Minimum	50.4	10000		47.9
PTV	Target	Maximum	50.5	8000		52.9
PTV	Target	Uniform dose	50.4	1000		50.4
Inner ears	OAR	Maximum average	33.0	200		50.0
Chiasma	OAR	Maximum	25.0	800		50.0
Pituitary	OAR	Maximum	30.0	800		30.0
Lens	OAR	Maximum	5.0	300		10.0
Optic nerve	OAR	Maximum	15.0	200		54.0
Bulbus oculi	OAR	*D*_50_%	5.0	200		35.0
Normal tissue	OAR	Dose falloff	50.4–33.0	20000	0.8	–
Normal tissue	OAR	Dose falloff	50.4–25.2	2000	1.6	–

### 2.B Dosimetry and peripheral low‐dose measurement

Dose verification of all IMRT and VMAT plans was performed with the MatriXX Evolution™ 2D ionization chamber array (IBA Dosimetry, Schwarzenbruck, Germany) positioned between a 10 cm (bottom) and a 9.7 cm (top) stack of RVW slabs (PTW, Freiburg, Germany). AAPM TG119 recommendations were used for the dose verification acceptance.^16^, ^17^ Gamma indices^18^ were calculated with a dose tolerance of 3% of the maximum dose and 3 mm distance to agreement and the gamma criterion was considered fulfilled if γ < 1 in at least 95% of the pixels.

**Figure 1:**
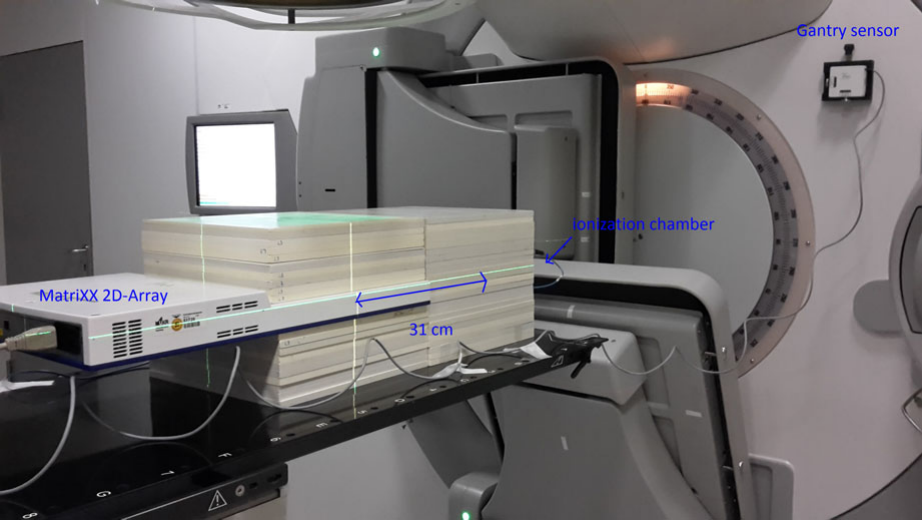
Measurement setup.

In the low‐dose region, the EAR presents a linear dose–response.^19^, ^20^ Since planning CT data do not include images at larger distances for reasons of radiation protection, no calculations of the EAR based on DVH are possible, for example, bladder, bowel, and gonads. Various studies have previously shown large uncertainties for dose calculations in the out‐of‐field area and therefore recommended to perform point dose measurements instead of calculations in this area.^9^, ^21^, ^22^ Therefore, we performed dose measurements in the same coronal plane in the low‐dose region at a distance of 31 cm cranial of the isocenter using a 0.3 ccm ionization chamber (PTW) simultaneously with 2D verification (Fig. 1). The target is symmetric in cranial‐caudal direction; therefore, the measurements can also be associated with the dose in the caudal region. The results were compared for the five irradiation techniques for the whole series. The complete setup is shown in Fig. 1.

Simultaneously, total delivery times were measured from first beam on to last beam off.

### 2.C Excess absolute risk calculation and organ equivalent dose

The excess absolute risk (EAR) to develop a solid cancer describes the absolute difference in cancer rates of persons exposed to a dose *d* and those not exposed to a dose beyond the natural dose exposition per 10,000 person‐year and is described as^23^

$$\mathrm{EAR}\left(D,e,a\right)=\mathrm{EA}R_{0}\;\mathrm{RED}\left(D\right)\;\mu \left(e,a\right)$$

where RED (risk equivalent dose) is the dose–response relationship for radiation‐induced cancer in units of dose and EAR_0_ the initial slope (slope of the dose–response curve at low dose). The function μ depends on the attained age *a* and age at exposure *e*:

$$\mu \left(e,a\right)=\exp(\gamma_{e}\left(a-30\right)+\gamma_{a}\ln\left(a/70\right))$$

For this study, the EAR values are calculated for gender‐averaged persons at an age at exposure of 10 yr and an attained age of 70 yr and reported per 10,000 person‐years. EAR_0_, γ_*e*_, and γ_*a*_ values have been derived by Preston et al.^20^ from the data of the Japanese atomic bomb survivors (EAR_0_ = 0.7 per 10,000 person‐years per Gy for the brain, γ_*e*_ **= −0.024, and γ_*a*_ **= 2.38). Large errors are involved in the determination of the parameters EAR_0_ and μ; therefore, absolute risk results have to be viewed with care.

It is known, that for doses below 2 Gy, the dose–response is linear.^8^ For higher doses, the concept of an organ equivalent dose (OED), which is proportional to EAR, has been defined by Schneider et al.^12^, ^13^:

$$\text{OED}=\frac{1}{V_{T}}\sum_{i}V\left(D_{i}\right)\mathrm{RED}\left(D_{i}\right)$$

Therefore, risk ratios for different treatment plans are equivalent to OED ratios which can be determined with the RED function and DVH.

There are different models for the RED calculation, based on different assumptions on the behavior of cells after dose exposition.^23^ The linear model assumes a linear response over the whole dose range:

RED(D)=D

The mechanistic model accounts for cell killing and fractionation effects^23^, ^24^:

$$\mathrm{RED}\left(D\right)=\frac{e^{-\alpha^{\prime }D}}{\alpha^{\prime }R}\left(1-2R+R^{2}e^{\alpha^{\prime }D}-{\left(1-R\right)}^{2}\right)e^{\frac{\left(-\alpha^{\prime }R\right)D}{1-R}}$$

where $\propto^{\prime }=\propto +\beta \frac{D}{D_{T}}d_{T}$

For this model, there are two limit cases: when the parameter *R* is 0 if no and 1 if full repopulation/repair occurs. Therefore, the limit *R* = 0 leads to the linear‐exponential model:

$$\mathrm{RED}\left(D\right)=Dexp\left(-\alpha^{\prime }D\right)$$

Moreover, the limit *R* = 1 is the case of full repopulation/repair, known as plateau model:

$$\mathrm{RED}\left(D\right)=\frac{1-\exp(-\alpha^{\prime }D)}{\alpha^{\prime }}$$

The parameters α and β are those from the linear quadratic model of cell killing. The parameter *R* describes the repopulation and repair ability between dose fractions. The parameters α′ = 0.018 and *R* = 0.93 for the brain have been derived from a combined fit to the data of atomic bomb survivors and Hodgkin patients treated with doses up to 40 Gy assuming that α/β = 3 Gy.^23^ α′ is 0.009 in case of no fractionation (linear‐exponential limit) and 0.021 in case of full tissue recovery (plateau limit).

As mentioned before, the parameters used in the calculation of absolute EAR involve large errors. In order to keep the uncertainties at minimum, we opted to use the OED values for evaluation of the risk of second cancer when comparing different radiation techniques. EAR absolute values were also calculated for the sake of completeness.

Previous studies have demonstrated that for inhomogeneous dose distributions above 4 Gy, the linear‐exponential, the plateau, and the full mechanistic model represent a better description of the dose–response function compared to the linear model.^12^, ^23^ Therefore, this model was not included in our results.

### 2.D Evaluation

Plan quality was assessed by the analysis of the DVH for the PTV and OARs. Target coverage was represented by the volume of the PTV covered by 95% of the prescription dose (*V*_95%_). The homogeneity index was defined as HI = (*D*_2%_ − *D*_98%_)/*D*_50%_, the conformity index^25^ as CI = *V*_95%_^2^/(TV·PIV). Here, TV means the volume of the PTV, PIV the total volume covered by 95% of the prescription dose. Relevant clinical parameters were evaluated for the OARs.

Differences between the irradiation modes were assumed to be clinically relevant when larger than the standard deviation within one group, which corresponds to an effect size of 1. For testing the two‐sided Wilcoxon signed‐rank test for matched pairs was used since this test does not require a normal parent distribution. Power analysis for the Wilcoxon test, however, requires assumptions for the parent distribution. Therefore, *a priori* power analysis according to Cohen^26^ was performed for various assumptions for the parent distribution to determine the required total sample size *N* for a statistical significance level of 0.05, a power of 0.8, and an effect size of 1 using the software G*Power version 3.1.9.2.^27^, ^28^ The calculated sample size was *N* = 11 for a normal parent distribution, *N* = 7 for a Laplace distribution, and *N* = 10 for a logistic distribution. A sample size of *N* = 11 was therefore chosen for this study.

The two‐sided Wilcoxon signed‐rank test implemented in IBM SPSS^®^ Statistics 23.0 (IBM Corporation) was used to detect relevant differences with a significance level of 0.05. Due to multiple pairwise comparisons,^29^ Holm–Bonferroni^30^ step‐down method for multiple testing was applied to control the maximum experimentwise error rate (MEER).^31^

## 3 RESULTS

**Figure 2:**
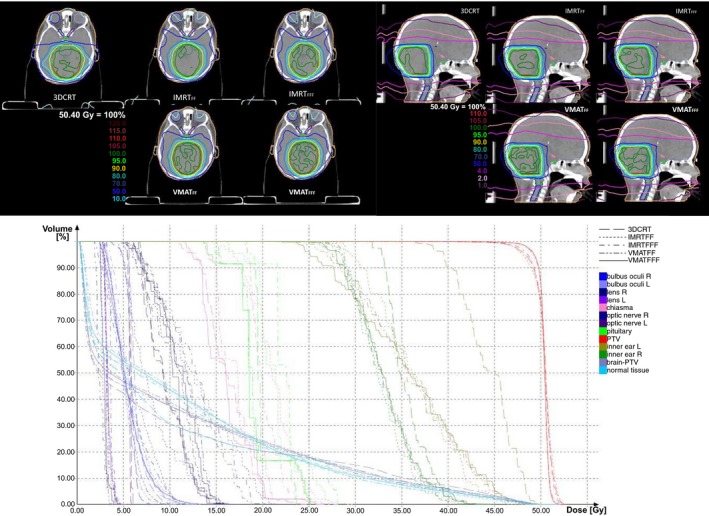
Comparison of dose distributions and DVH for a typical case in a transversal and sagittal slice.

A comparison of dose distributions and DVH for all techniques and modes is shown in Fig. 2 for one typical case. Isodoses are shown for the range of 10–110% of 50.4 Gy. In the sagittal slice, the low‐dose region is represented for the range of 1–4% for better comparison of the dose distribution in low‐dose region.

Plan quality was evaluated by means of the DVH and dose distributions. Details of relevant DVH parameters are given in Table 2 averaged over all patients. Threshold doses listed in Table 1 were met in all 55 plans. FFF modes led to significantly higher CI and HI of the target compared to 3DCRT. IMRT_FFF_ led to a significantly superior target CI, HI, and *V*_95%_. No significant differences were found between VMAT_FFF_ and VMAT_FF_ in the PTV. All dose–volume parameters evaluated for the OARs were similar without significant differences between IMRT and VMAT (comparing FF and FFF modes). The comparison of IMRT_FFF_/VMAT_FFF_ with the standard plan 3DCRT led to a significant lower dose for the inner ears, right lens, and bulbus oculi when using FFF. On the contrary, in the structure brain‐PTV and normal tissue, comparison of FFF modes with 3DCRT led to a significantly lower dose when 3DCRT was used as treatment technique. When comparing FFF with FF in IMRT and VMAT, the evaluated parameter in low‐dose area *V*_4Gy_ in the normal tissue was significantly lower when using FFF.

**Table 2:** Treatment plan comparison

		3DCRT	IMRT_FF_	IMRT_FFF_	VMAT_FF_	VMAT_FFF_
PTV	*V*_95%_ (%)	97.2 ± 0.7	96.3 ± 0.8	97.7 ± 0.7^**s**^	99.1 ± 0.6	**99.0 ± 0.7**
	HI	0.09 ± 0.01	0.09 ± 0.01	**0.08 ± 0.01** ^s^	0.06 ± 0.01	**0.06 ± 0.01**
	CI	0.84 ± 0.06	0.90 ± 0.04	**0.89 ± 0.04** ^s^	0.86 ± 0.04	**0.87 ± 0.04**
Inner ear L	*D*_50%_	35.7 ± 7.2	30.9 ± 4.3	**30.5 ± 4.1**	31.4 ± 4.6	**31.0 ± 4.6**
Inner ear R	*D*_50%_	33.8 ± 8.0	28.7 ± 6.9	**28.7 ± 6.3**	29.5 ± 5.4	**29.6 ± 6.1**
Chiasma	*D*_2%_	23.6 ± 8.7	24.0 ± 4.6	24.2 ± 3.9	23.9 ± 4.7	24.2 ± 4.3
Pituitary	*D*_2%_	23.9 ± 8.9	24.1 ± 6.2	24.7 ± 5.9	23.9 ± 4.9	23.8 ± 5.1
Lens L	*D*_max_	4.0 ± 2.4	3.8 ± 2.2	3.7 ± 2.1	3.6 ± 1.6	3.6 ± 1.9
Lens R	*D*_max_	4.7 ± 2.7	3.6 ± 1.9	**3.6 ± 2.0**	3.6 ± 1.6	**3.4 ± 1.8**
Optic nerve L	*D*_max_	13.9 ± 3.7	16.5 ± 3.1	16.6 ± 3.3	15.4 ± 0.8	14.9 ± 2.0
Optic nerve R	*D*_max_	14.6 ± 3.1	16.7 ± 2.8	16.7 ± 3.7	15.3 ± 1.3	15.7 ± 0.9
Bulbus oculi L	*D*_mean_	3.9 ± 2.2	4.2 ± 2.3	4.2 ± 2.3	4.2 ± 1.9	4.2 ± 2.2
Bulbus oculi R	*D*_mean_	4.7 ± 2.7	4.1 ± 2.3	4.1 ± ± 2.3	4.1 ± 1.9	**3.9** ± **2.0**
Brain‐PTV	*D*_50%_	**4.5 ± 2.3**	6.2 ± 4.1	6.0 ± 4.1	6.7 ± 3.8	6.4 ± 4.0
	*D*_mean_	**11.1 ± 1.0**	11.6 ± 1.8	11.4 ± 1.8	12.3 ± 1.6	12.0 ± 1.8^s^
Normal tissue	*D*_50%_	**3.4 ± 1.8**	4.0 ± 2.1	3.9 ± 2.0	4.8 ± 2.1	4.4 ± 2.9^s^
	*V*_4 Gy_ (%)	**46.9 ± 6.4**	48.5 ± 6.7	48.0 ± 6.8^s^	51.0 ± 6.3	49.9 ± 6.5^**s**^

Values are averaged over all patients; standard deviation is given for all values. All values are given in Gy except *V*_95%_ and *V*_4Gy_ in percentage of the structure volume. L and R indicate left and right. Values highlighted in bold are statistically significant when comparing IMRT_FFF_ with 3DCRT and VMAT_FFF_ with 3DCRT. s indicates that the value is statistically significant when comparing both IMRT modes and both VMAT modes. For the PTV, conformity index (CI) and homogeneity index (HI) were evaluated: CI = (TV_95%_)^2^/(TV·V_95%_), where TV_95%_ is the target volume covered by the 95% isodose, TV is the target volume, and *V*_95%_ is the volume of the reference isodose; HI = (*D*_2%_ − *D*_98%_)/*D*_50%_.

Since no plan exceeded the threshold values, all plans achieved acceptable plan quality in terms of dose sparing in the OARs.

### 3.A Dosimetry and delivery

Gamma evaluation of the verification of dose calculation is shown in Table 3. All 44 IMRT and VMAT plans passed the evaluation with passing rates >95% as recommended by the AAPM TG119.^16^, ^17^ On average, IMRT_FFF_ achieved a higher gamma passing rate than the other techniques, but no significant differences were found.

**Table 3:** Total delivery time, peripheral dose (D_IC_) and gamma passing rate of the dose verification

	3DCRT	IMRT_FF_	IMRT_FFF_	VMAT_FF_	VMAT_FFF_
Time	**142 ± 9**	293 ± 43	288 ± 32	72 ± 3	**72 ± 7**
D_IC_	32.78 ± 4.18	40.67 ± 6.41	**28.12** ^s^ ** ± 5.35**	34.54 ± 3.03	**24.67** ^s^ ** ± 3.44**
Gamma passing rate	–	97.84 ± 1.47	99.02 ± 0.99	97.05 ± 1.24	97.38 ± 1.01

Total delivery time measured in seconds. D_IC_ in mGy measured with an ionization chamber at 31 cm of isocenter for 28 fractions. Values highlighted in bold are statistically significant when comparing IMRT_FFF_ with 3DCRT and VMAT_FFF_ with 3DCRT. S indicates that the value is statistically significant when comparing both IMRT modes and both VMAT modes.

Total delivery times are also listed in Table 3. Both VMAT modes reduced the delivery time significantly (*P* = 0.003) by 50% compared to 3DCRT. Both IMRT modes doubled significantly the delivery time compared to 3DCRT (*P* = 0.003).

Peripheral dose was measured with an ionization chamber in the measurement setup of Fig. 1; the measured doses are listed in Table 3. It was observed that FFF reduced the peripheral dose significantly compared with FF techniques for IMRT and VMAT techniques as well as for 3DCRT. The significantly lowest peripheral dose was found for VMAT_FFF_.

### 3.B Excess absolute risk of developing secondary cancer and organ equivalent dose

Table 4 shows the calculated OED values for the linear‐exponential, plateau, and full mechanistic dose–response model. 3DCRT was the technique with the lowest OED values. When comparing 3DCRT with VMAT_FF/FFF_, the advantage of 3DCRT was significant in all three models, whereas the 3DCRT advantage over IMRT_FF/FFF_ was found not significant. No significant differences were found between IMRT_FF/FFF_, whereas VMAT_FFF_ values were significantly lower than VMAT_FF_. IMRT_FF_ could achieve significant lower OED values when compared with VMAT_FF_. The same behavior was observed for IMRT_FFF_/VMAT_FFF_.

**Table 4:** OED in the brain and standard deviation

Plan	OED_lin‐exp_	OED_plateau_	OED_mech_
3DCRT	*8.60 ± 0.85*	*8.41 ± 0.83*	*7.58 ± 0.78*
IMRT_FF_	9.09* ± 1.51	8.89* ± 1.48	8.08* ± 1.39
IMRT_FFF_	8.99* ± 1.56	8.79* ± 1.53	7.98* ± 1.43
VMAT_FF_	9.67 ± 1.40	9.46 ± 1.37	8.58 ± 1.29
VMAT_FFF_	**9.41 ± 1.52**	**9.20 ± 1.50**	**8.34 ± 1.40**

Values in italic are significant when comparing both VMAT_FF/FFF_ with 3DCRT, bold indicates that the value is statistically significant when comparing VMAT_FFF_ to VMAT_FF_, *indicates statistically significant when comparing IMRT_FF_ with VMAT_FF_ and IMRT_FFF_ with VMAT_FFF_.

For completeness, EAR values are listed in Table 5. The values are proportional to OED; no statistical analysis of the data has been performed because of the large uncertainties of the factors EAR_0_ and μ used in the calculation.

**Table 5:** Excess absolute risk (EAR) for brain

Plan	EAR_lin‐exp_	EAR_plateau_	EAR_mech_
3DCRT	9.73	9.51	8.58
IMRT_FF_	10.28	10.06	9.15
IMRT_FFF_	10.17	9.94	9.03
VMAT_FF_	10.94	10.70	9.71
VMAT_FFF_	10.65	10.41	9.43

## 4 DISCUSSION

The main objective of this study is to investigate the potential of the FFF mode of a linear accelerator to reduce the radiation‐induced second cancer risk, the treatment time, and to improve the treatment plan quality in pediatric ependymoma.

The major concern of using inverse treatment planning for the treatment of ependymoma is that the high‐dose falloff at the margin of the PTV might lead to a decrease of the local tumor control. However, recent publications for ependymoma showed that the local control and overall survival achieved with IMRT are not inferior to those associated with conventional therapy.^4^, ^32^, ^33^ We performed a DVH comparison of five different techniques: 3DCRT, IMRT_FF/FFF_, and VMAT_FF/FFF_ to determine if target coverage or OAR dose sparing is being compromised using inverse techniques. The results indicate that, for all IMRT and VMAT techniques, the homogeneity and conformity indexes are similar or even improved significantly (FFF mode) as compared to 3DCRT. Regarding the OARs, since all threshold values were fulfilled, we assume that inverse techniques do not affect the plan quality. These results indicate that the local tumor control would not be compromised using inverse techniques.

The total delivery time becomes an important issue when treating pediatric patients. Our study showed that VMAT represents an advantage for treating those patients. In both irradiation modes, FF as well as FFF, VMAT could reduce the delivery time to the half compared to 3DCRT. An additional advantage of FFF over FF could not be observed. This can be explained by the fact that the maximum dose rate was not exploited in VMAT_FFF_, since the minimum time of 1 minute required for one rotation was already nearly achieved in FF mode, as it has already been mentioned in a previous study for hypopharynx carcinoma.^34^

Pediatric patients are particularly vulnerable to radiation exposure. The peripheral dose in pediatric radiation has been previously investigated by Mansur et al.^35^ Their investigation showed that the peripheral dose at large distances using 3DCRT was lower than using IMRT. Since planning CT do not include the peripheral region, no calculations of the second cancer risk based on DVH are possible. Various studies have previously shown large uncertainties for dose calculations in the out‐of‐field area and therefore recommended to perform point dose measurements instead of calculations in this area.^9^, ^21^, ^22^, ^36^ Therefore, peripheral dose measurements were performed and compared at a distance of 31 cm to the isocenter. These measurements can be associated with the location of peripheral organs such as gonads, bladder, bowel, or stomach, depending on the size of the patient. The results of our study are comparable with the study mentioned above. No significant differences could be observed in peripheral dose between VMAT/IMRT_FF_ and 3DCRT. However, removing the flattening filter allows a significant reduction of the peripheral doses compared to 3DCRT. The peripheral dose reduction was found to be 15% in case of IMRT_FFF_ and 25% in case of VMAT_FFF_. Due to the linearity of the dose–response for doses up to 2 Gy, the EAR in organs situated at large distances of the PTV is proportional to D_IC_. Therefore, the EAR to organs in the region of the peripheral dose measurement could be reduced by 15% for IMRT_FFF_ and 25% for VMAT_FFF_. To calculate the OED and EAR in the tissue near the PTV, three different dose–response models have been calculated based on DVH data of the structure brain‐PTV. The dose calculation performed with Oncentra External Beam^®^4.5.2 was verified with a 2D array to assure that the DVHs can be used for calculating the EAR. Although the study demonstrated that the use of flattening filter free beams reduces the peripheral dose at large distances, this could not be translated into a general reduction of the second cancer risk for structures near to the tumor. The FFF mode allowed a significant reduction as compared to the flat beam mode for VMAT. For IMRT, no significant differences were found between both irradiation modes. The lowest EAR of all plans was, however, achieved with 3DCRT.

The results of our study did not show any advantage of using IMRT_FF_/VMAT_FF_ over the standard 3DCRT technique. IMRT_FFF_ allowed reducing the peripheral dose compared to 3DCRT, but the irradiation time was significantly higher compared to other techniques. For this reason, it would not be recommended for treating pediatric patients. For VMAT_FFF_, a significant reduction of the peripheral dose and irradiation time was observed.

The Holm–Bonferroni step‐down method for multiple testing was applied to the comparison of HI, CI, time, EAR_mech_, and D_IC_ for 3DCRT vs V_FFF_ to control MEER. No hypothesis had to be rejected; therefore, all significant results remained significant after the test.

## 5 CONCLUSION

The results of this study show that VMAT_FFF_ is a technique to consider for treating childhood ependymoma. Compared to the standard technique used for ependymoma, VMAT_FFF_ achieved significantly higher PTV coverage and allowed reducing treatment time significantly. The risk of radiation‐induced secondary cancer, which is a major concern in radiation therapy of pediatric patients, was significantly reduced for organs located at large distance from the target, for example, bladder, gonads, or bowel. No advantage could be observed with respect to the secondary cancer risk in the brain.

## CONFLICT OF INTEREST

The authors declare that there is no conflict of interest regarding the publication of this article.
